# The long noncoding RNA HCG18 participates in PM2.5-mediated vascular endothelial barrier dysfunction

**DOI:** 10.18632/aging.104073

**Published:** 2020-11-16

**Authors:** Shiwen Wang, Yuyin Lin, Yue Zhong, Maozhou Zhao, Wei Yao, Xiaohui Ren, Qin Wang, Xiaolan Guo, Qian-Qian Zhang, Jianwei Dai

**Affiliations:** 1 GMU-GIBH Joint School of Life Sciences, Center of Reproductive Medicine, Third Affiliated Hospital, Guangzhou Medical University, Guangzhou 510182, China; 2 The State Key Lab of Respiratory Disease, Guangzhou Institute of Respiratory Disease, The First Affiliated Hospital, Guangzhou Medical University, Guangzhou 510120, China; 3 Vascular Biology Research Institute, School of Basic Course, Guangdong Pharmaceutical University, Guangzhou 510006, China; 4 Healthcare Department, Agency for Offices Administration, Haidian District, Beijing 100082, China; 5 Ocean College of Hebei Agricultural University, Qinhuangdao 066003, China; 6 Key Laboratory for Reproductive Medicine of Guangdong Province, Key Laboratory for Major Obstetric Disease of Guangdong Province, Key Laboratory of Reproduction and Genetics of Guangdong Higher Education Institutes, Third Affiliated Hospital of Guangzhou Medical University, Guangzhou 510150, China; 7 National Institute of Environmental Health, Chinese Center for Disease Control and Prevention, Beijing 100021, China

**Keywords:** HCG18, PM2.5, SOX7, vascular endothelial cell, permeability

## Abstract

Increased vascular endothelial permeability can disrupt vascular barrier function and further lead to multiple human diseases. Our previous reports indicated that particulate matter 2.5 (PM2.5) can enhance the permeability of vascular endothelial cells. However, the regulatory mechanism was not comprehensively demonstrated. Therefore, this work elucidated this mechanism by demonstrating that PM2.5 can increase the permeability of HUVECs by inhibiting the expression of Hickson compact group 18 (HCG18). Moreover, we demonstrated that lncRNA HCG18 functioned as a ceRNA for miR-21-5p and led to the derepression of its target SOX7, which could further transcriptionally activate the expression of VE-cadherin to regulate the permeability of HUVECs. In this study, we provide evidence that HCG18/miR-21-5p/SOX7/VE-cadherin signaling is involved in PM2.5-induced vascular endothelial barrier dysfunction.

## INTRODUCTION

The blood vessel wall forms a selective semipermeable barrier to regulate the exchange of macromolecules between the blood and tissues. Vascular endothelial cells are located in the blood vessel wall and play a critical role in vascular homeostasis by maintaining endothelial barrier function [1]. Disruption of the vascular barrier can lead to multiple human diseases, such as lung disease, cancer, angiocardiopathy, ischemia, and oculopathy [1–4]. Endothelial permeability involves the transcellular and paracellular pathways. Blood components can pass through individual endothelial cells by the transcellular pathway, which maintains vascular homeostasis in the physiological state [2, 5]. However, vascular leakage usually occurs in pathological conditions and results in blood components crossing intercellular cell-to-cell junctions by the paracellular pathway [2, 5]. Vascular leakage is often initiated by various external stimuli, including the release of vascular endothelial growth factor (VEGF), thrombin, or histamine and the activation of leukocytes [2]. In addition, our previous report indicated that ambient particulate matter with an aerodynamic diameter <2.5 μm (PM2.5) can disrupt vascular endothelial barrier function by damaging vascular endothelial cells [6, 7]. Vascular endothelial (VE)-cadherin is critical for endothelial adherent junctions [8, 9]. Our previous report indicated that PM2.5 can increase vascular endothelial permeability through miR-21-5p, indirectly inhibiting the expression of VE-cadherin [6]. However, the regulatory mechanism between miR-21-5p and VE-cadherin is still undetermined.

Long noncoding RNAs (lncRNAs), a class of nonprotein coding transcripts that exceed 200 nucleotides in length, have low sequence conservation and high tissue specificity [10, 11]. Accumulating evidence has shown that lncRNAs function as important regulators that are widely involved in various physiological and pathological processes, including development, stem cell differentiation, and tumorigenesis [12–15]. Although a large number of lncRNAs have been identified, very few have a characterized biological function. Therefore, the potential functional characterization of lncRNAs in biological processes has attracted much attention. LncRNAs can modify gene expression at the transcriptional, posttranscriptional or translational level. Recently, a new regulatory mechanism has been clarified, in which lncRNAs function as competing endogenous RNAs (ceRNAs) to regulate gene expression by sponging microRNAs (miRNAs) [16]. Hickson compact group 18 (HCG18) is a lncRNA, and its potential biological function has not been well elucidated. It has been shown that HCG18 acts as a ceRNA of miR-146-5p and is up-regulated in intervertebral disc degeneration [11]. However, Xu et al. demonstrated that HCG18 is down-regulated in bladder cancer tissues and increases NOTCH1 expression by competitively sponging miR-34-5p [17]. Therefore, the cell type-dependent function and the regulatory molecular mechanisms of HCG18 remain largely unknown.

Our previous study demonstrated that miR-21-5p is involved in PM2.5-induced endothelial permeability increase by indirectly inhibiting VE-cadherin expression [6]. We hereby aimed to explore the potential lncRNA that could sponge miR-21-5p during PM2.5-mediated vascular endothelial barrier dysfunction and the regulatory mechanism between miR-21-5p and VE-cadherin. First, microarray analysis was utilized to identify the lncRNA HCG18 that might be involved in PM2.5-induced miR-21-5p overexpression. Next, we used luciferase reporter assays and RNA immunoprecipitation (RIP) to confirm that HCG18 functions as a competing endogenous RNA (ceRNA) by sponging miR-21-5p. Then, we found that SOX7 is a target of miR-21-5p and that it can transcriptionally regulate VE-cadherin expression. Our findings suggest that inhibition of HCG18 promotes PM2.5-mediated vascular endothelial barrier dysfunction by sponging miR-21-5p.

## RESULTS

### LncRNA HCG18 might function as a candidate miR-21-5p sponge and is down-regulated by PM2.5 in vascular endothelial cells

**Figure 1:**
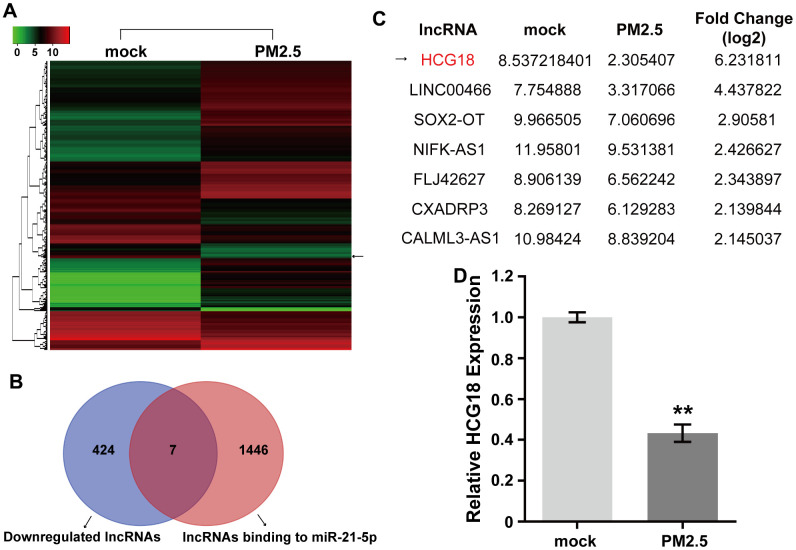
**PM2.5 inhibits the expression of HCG18 in HUVECs.** (**A**) The differentially expressed lncRNAs between PM2.5-treated and untreated HUVECs were detected using lncRNA microarray. (**B**) Venn diagram shows the overlap between down-regulated lncRNAs in PM2.5-treated HUVECs and predicted lncRNAs binding to miR-21-5p. (**C**) HCG18 was the most down-regulated lncRNA among the 7 PM2.5-regulated lncRNAs. (**D**) The difference of HCG18 level between PM2.5-treated and untreated HUVECs was confirmed by qRT-PCR. **, *P*<0.01.

Our previous report indicated that exposure to PM2.5 can induce vascular endothelial barrier dysfunction through STAT3-dependent up-regulation of miR-21-5p. LncRNAs can act as endogenous miRNA sponges by competitively binding to miRNAs and suppressing their expression and function [18]. To further investigate the regulatory mechanism involved in PM2.5-induced miR-21-5p overexpression during the increase in vascular permeability, the differentially expressed lncRNAs between PM2.5-treated and untreated HUVECs were detected using lncRNA microarray. The microarray data showed that 431 lncRNAs were down-regulated and 910 lncRNAs were up-regulated in PM2.5-treated HUVECs compared with the untreated group (Figure 1A). Furthermore, the possible lncRNAs that could bind to miR-21-5p were analyzed using a Venn diagram generated by an online tool (http://bioinformatics.psb.ugent.be/webtools/Venn/). Analysis of the lncRNA microarray results and Venn diagram identified 1453 candidate lncRNAs as functional ceRNAs that could bind to miR-21-5p; however, only 7 lncRNAs were significantly down-regulated by PM2.5 in HUVECs (Figure 1B). In addition, HCG18 was the most significantly down-regulated lncRNA among these 7 PM2.5-regulated lncRNAs predicted to bind to miR-21-5p (Figure 1C). Furthermore, the down-regulation of HCG18 by PM2.5 was confirmed by qRT-PCR in HUVECs (Figure 1D).

### LncRNA HCG18 acts as a ceRNA for miR-21-5p to form a HCG18/miR-21-5p axis and is regulated by PM2.5 in HUVECs

**Figure 2:**
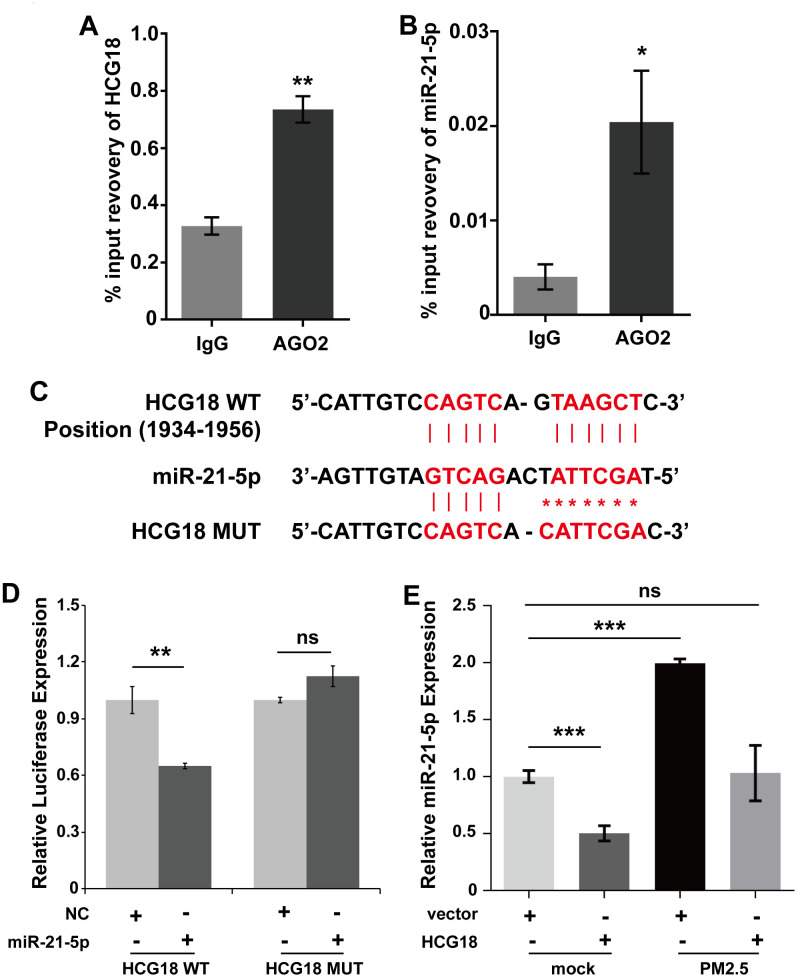
**LncRNA HCG18 acts as a ceRNA for miR-21-5p.** (**A**) HCG18 was enriched in AGO2 RIP samples. (**B**). MiR-21-5p was enriched in AGO2 RIP samples. (**C**) The binding relationship between HCG18 and miR-21-5p was predicted by DIANA. (**D**) The binding relationship between HCG18 and miR-21-5p was determined by Dual-luciferase reporter assay. (**E**) The interaction between HCG18 and miR-21-5p in PM2.5-treated HUVECs was analyzed by qRT-PCR. *: *P*<0.05; **: *P*<0.01; ***: *P*<0.001.

To further confirm that HCG18 can sponge miR-21-5p to inhibit its function, a RIP assay using an antibody against human AGO2 was used. Ago2 protein-RNA complexes in HUVECs were precipitated by the Ago2 antibody, and then the expression of HCG18 and miR-21-5p was detected using qRT-PCR. We found that endogenous HCG18 and miR-21-5p were both enriched in AGO2 RIP samples compared with control IgG

antibody RIP samples from HUVECs (Figure 2A, 2B). These results indicate that HCG18 and miR-21-5p are in the same AGO2 complex in HUVECs. Then, the binding relationship between HCG18 and miR-21-5p was further investigated. The predicted miR-21-5p binding sites within HCG18 were analyzed using the DIANA tool (http://carolina.imis.athena-innovation.gr/diana_tools/web/index.php) (Figure 2C). To further verify that HCG18 directly targets miR-21-5p, we performed luciferase reporter assays. The results revealed that miR-21-5p mimics markedly suppressed the luciferase activity of wild-type HCG18 (HCG18-WT) but not that of mutant HCG18 (HCG18-MUT) (Figure 2D). Together, these data suggest that HCG18 functions as a ceRNA for miR-21-5p in HUVECs. Next, we further clarified whether PM2.5 regulates the HCG18/miR-21-5p axis in HUVECs. The interaction between HCG18 and miR-21-5p in PM2.5-treated HUVECs was analyzed by qRT-PCR. The results showed that overexpression of HCG18 significantly inhibited miR-21-5p; however, the induction of miR-21-5p by PM2.5 could be abolished by overexpression of HCG18 in HUVECs (Figure 2E). Overall, we confirmed that PM2.5 can disrupt the balance of competitive binding between HCG18 and miR-21-5p in HUVECs.

### LncRNA HCG18 functions as a ceRNA to enhance SOX7 expression by competitively sponging miR-21-5p in PM2.5-treated vascular endothelial cells

**Figure 3:**
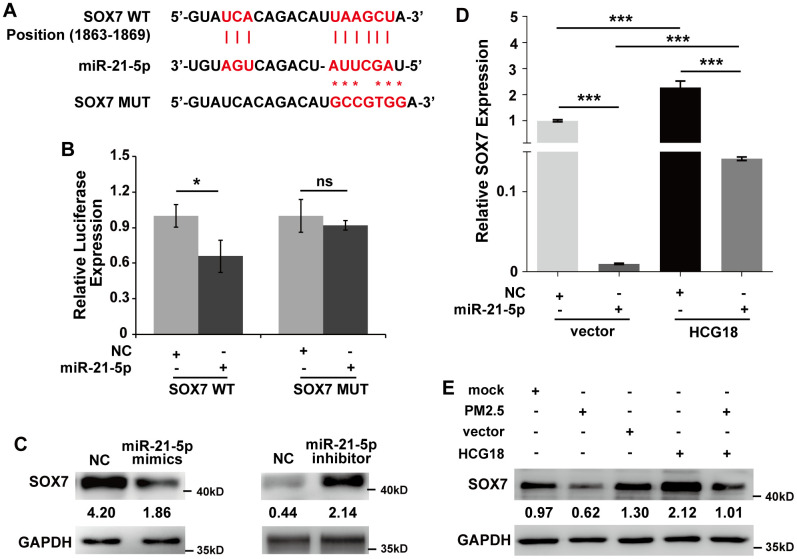
**LncRNA HCG18 functions as a ceRNA to enhance SOX7 expression by competitively sponging miR-21-5p in PM2.5-treated HUVECs.** (**A**) The binding relationship between miR-21-5p and SOX7 3’UTR was predicted online. (**B**) Dual-luciferase assays indicated that the expression of SOX7 was inhibited by miR-21-5p. (**C**) The protein expression of SOX7 regulated by miR-21-5p was detected using Western blotting. (**D**) The mRNA level of SOX7 was decreased by miR-21-5p and increased by overexpression of HCG18. Moreover, miR-21-5p abrogated the up-regulation of SOX7 in HUVECs overexpressing HCG18. (**E**) The data of Western blotting showed that PM2.5 suppressed SOX7 expression, and HCG18 positively regulated SOX7 expression. In addition, the decreased expression of SOX7 by PM2.5 could be reversed by overexpression of HCG18. *: *P*<0.05; ***: *P*<0.001.

It has been demonstrated that a single miRNA can play different biological functions by regulating the expression of multiple target genes. To confirm whether miR-21-5p regulates the expression of other signaling molecules to increase the permeability of vascular endothelial cells, we further analyzed candidate target genes that play important roles in the regulation of vascular barrier function and might be regulated by miR-21-5p. As shown in Figure 3A, the transcription factor SOX7 possessed one binding site for miR-21-5p in its 3’UTR. Next, a luciferase reporter assay was used to determine whether SOX7 is a direct target of miR-21-5p. The activity of the luciferase reporter carrying the wild-type putative target site of SOX7 was significantly inhibited by miR-21-5p compared with the negative control; however, the inhibitory effect was abrogated when mutations were introduced into the seed sequences of SOX7 complementary to miR-21-5p (Figure 3B).

In addition, the endogenous protein expression of SOX7 regulated by miR-21-5p was detected using western blotting. The results showed that SOX7 was significantly inhibited by overexpression of miR-21-5p but was increased by suppression of miR-21-5p in HUVECs (Figure 3C). Simultaneously, we further clarified whether HCG18 regulated miR-21-5p/SOX7 signaling to form a ceRNA pathway in HUVECs. To this end, we detected the gene expression of SOX7 by qRT-PCR assay. As shown in Figure 3D, the mRNA level of SOX7 was significantly decreased by miR-21-5p and markedly increased by overexpression of HCG18 in HUVECs. Nevertheless, cotreatment with miR-21-5p and HCG18 abrogated the upregulation of SOX7 mRNA in HUVECs overexpressing HCG18 (Figure 3D). Overall, the HCG18/miR-21-5p/SOX7 axis could form a ceRNA pathway in HUVECs. Next, we further determined whether PM2.5 regulates the HCG18/miR-21-5p/SOX7 ceRNA pathway in HUVECs. The results showed that PM2.5 clearly suppressed SOX7 expression, and HCG18 positively regulated SOX7 expression in HUVECs (Figure 3E). In addition, the decreased expression of SOX7 by PM2.5 could be reversed by overexpression of HCG18 in HUVECs (Figure 3E). These data further confirmed that the HCG18/miR-21-5p/SOX7 pathway was involved in the regulatory effect of PM2.5 in HUVECs.

### The lncRNA HCG18/miR-21-5p/SOX7/VE-cadherin axis is regulated by PM2.5 in vascular endothelial cells

**Figure 4:**
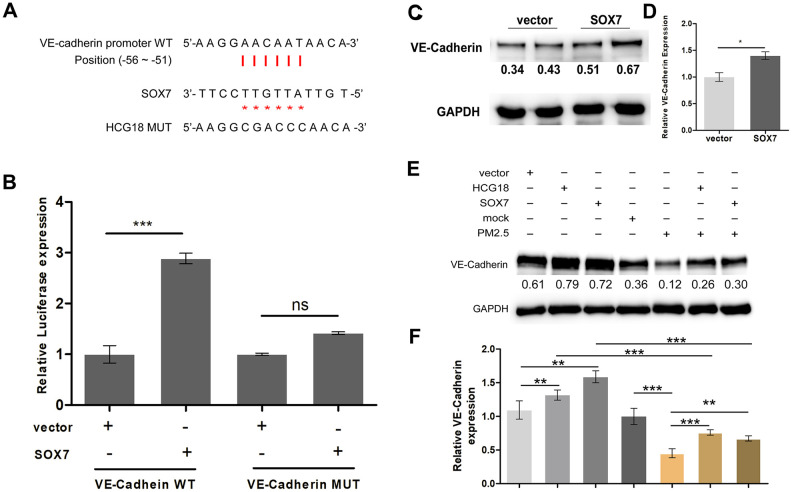
**The lncRNA HCG18/miR-21-5p/SOX7/VE-cadherin axis is regulated by PM2.5 in vascular endothelial cells.** (**A**) The binding relationship of SOX7 and the promoter region of VE-cadherin was predicted online. (**B**) Dual-luciferase reporter assay showed that SOX7 promote the transcriptional activities of VE-cadherin. (**C**) The protein expression of VE-cadherin was up-regulated in SOX7-overexpressed HUVECs. (**D**) The quantitative analysis of the results of C. (**E**) Overexpression of HCG18 promoted the expression of VE-cadherin, and the induction effect was abrogated by both overexpression of HCG18 or SOX7 and treatment with PM2.5. (**F**) The quantitative analysis of the results of E. ns: no significantly different. ***: *P*<0.001.

It has been demonstrated that SOX7 can transcriptionally activate mouse VE-cadherin in the hemogenic endothelium [19]. However, whether SOX7 regulates human VE-cadherin transcription by binding to the promoter region remains to be further confirmed. Our previous report indicated that miR-21-5p could indirectly inhibit the expression of VE-cadherin in HUVECs [6]. Therefore, we speculated that HCG18/miR-21-5p/SOX7 signaling may target VE- cadherin in PM2.5-treated vascular endothelial cells. First, a conserved SOX binding site from position -56 to -51 was found in the human VE-cadherin promoter (Figure 4A). We then performed a luciferase reporter assay to detect whether SOX7 could directly act on the promoter region. The SOX7 expression plasmid and the VE-cadherin promoter reporter vector, which included the SOX binding sites, were cotransfected into HEK293T cells. As shown in Figure 4B, the luciferase levels were increased 3-fold by the presence of SOX7, and the effect was abolished when the binding sites in the promoter region were mutated. We further detected the protein expression of VE-cadherin using western blotting and found that the expression of VE-cadherin was significantly upregulated when SOX7 was overexpressed in HUVECs (Figure 4C). In addition, overexpression of HCG18 promoted the expression of VE-cadherin, and the induction effect was abrogated by both overexpression of HCG18 or SOX7 and treatment with PM2.5 (Figure 4E). Together, these results show that SOX7 can transcriptionally regulate the expression of VE-cadherin, and HCG18/miR-21-5p/SOX7/VE-cadherin signaling is regulated by PM2.5 in HUVECs.

### PM2.5 increased the permeability of vascular endothelial cells through regulation of the HCG18/miR-21-5p/SOX7/VE-cadherin axis

**Figure 5:**
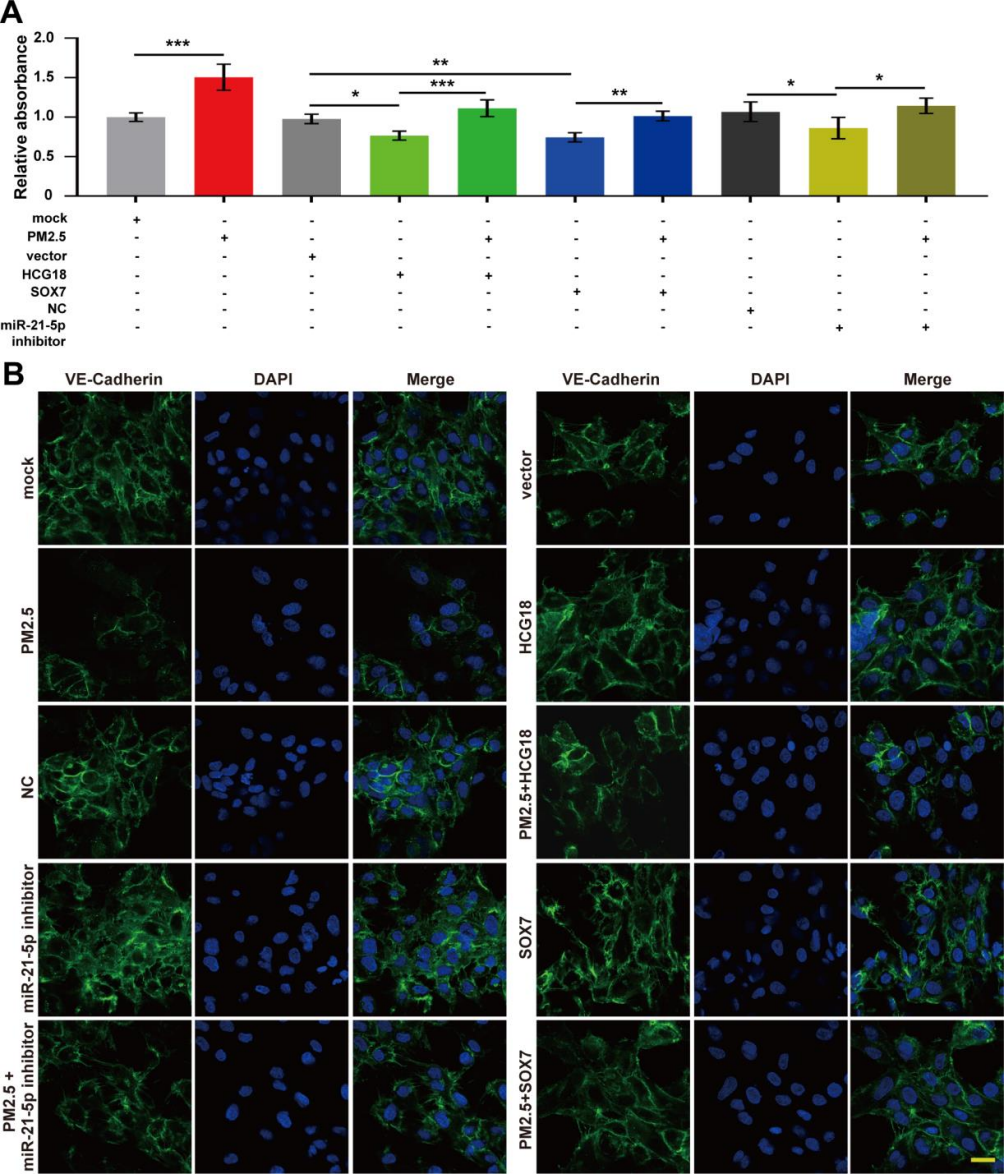
**PM2.5 increased the permeability of vascular endothelial cells through regulation of the HCG18/miR-21-5p/SOX7/VE-cadherin axis.** (**A**) The permeability detected by FITC-dextran transwell assay increased in PM2.5-treated HUVECs and decreased in HCG18 or SOX7-overexpressed or miR-21-5p-inhibited HUVECs. (**B**) Immunofluorescence assay was used to detect the expression and location of VE-cadherin in HUVECs. The expression of VE-cadherin was inhibited in PM2.5-treated HUVECs and increased in HCG18 or SOX7-overexpressed or miR-21-5p-inhibited HUVECs. Cotreatment with PM2.5 abrogated the overexpression of VE-cadherin induced by overexpression of HCG18 or SOX7 or inhibition of miR-21-5p. *: *P*<0.05; **: *P*<0.01; ***: *P*<0.001. Scale bar: 50 μm.

According to our findings above, whether HCG18 regulates vascular endothelial barrier dysfunction is still unknown. As shown in Figure 5A, treatment with PM2.5 significantly increased the permeability of HUVECs compared with that of the untreated group. In addition, overexpression of HCG18 and SOX7 or inhibition of miR-21-5p significantly inhibited FITC-dextran diffusion across the HUVEC sheet (Figure 5A). Moreover, the effect of HCG18, SOX7 and miR-21-5p inhibitor on the permeability of HUVEC monolayers was abrogated by cotreatment with PM2.5 (Figure 5A). Then, we stained the cells with VE-cadherin to visualize adherent junctions and found that VE-cadherin staining tended to be localized at cell-cell junctions. Furthermore, treatment with PM2.5 significantly inhibited the expression of VE- cadherin in treated HUVECs compared with the untreated group. In addition, overexpression of HCG18 or SOX7 or inhibition of miR-21-5p significantly increased the expression of VE-cadherin compared with that in the control groups (Figure 5B). Cotreatment with PM2.5 abrogated the overexpression of VE-cadherin induced by overexpression HCG18 or SOX7 or inhibition of miR-21-5p. (Figure 5B). Together, these results showed that PM2.5 may increase the permeability of HUVECs by regulating HCG18/miR-21-5p/SOX7/VE-cadherin signaling.

## DISCUSSION

**Figure 6:**
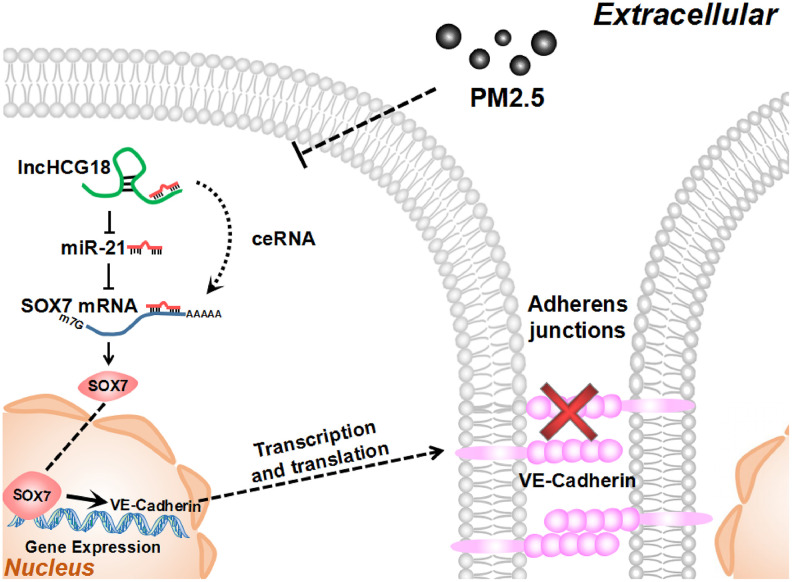
**PM2.5 increased permeability of HUVECs through HCG18/miR-21-5p/SOX7/VE-cadherin signaling.**

This study demonstrates that down-regulation of HCG18 results in vascular endothelial barrier dysfunction and functions as a ceRNA that is involved in PM2.5-induced miR-21-5p expression. These data provide support for a model in which HCG18 is down-regulated upon exposure to PM2.5 in vascular endothelial cells, which directly results in the up-regulation of miR-21-5p through the function of HCG18 as a ceRNA. High levels of miR-21-5p inhibit the expression of its target gene SOX7, which could bind to the VE-cadherin promoter region to regulate its transcriptional activity. In addition, constitutive suppression of the expression of VE-cadherin disrupts the junctions of endothelial cells and results in vascular endothelial barrier dysfunction (Figure 6).

In the past few years, it has become clear that the transcriptome is far larger and more complex than previously thought. The protein-coding genes account for 2% of the genome, while most of the transcripts do not encode any protein and were considered as “junk RNA” for a long time [20–22]. Recent investigations have found that noncoding RNAs (ncRNAs) play important regulatory roles in various physiological and pathological processes. Both miRNAs and lncRNAs are defined as functional ncRNAs in the transcriptome. miRNAs are short and highly conserved, and their functions have been well defined in recent years. Although there are some reports about the role of lncRNAs in cellular processes, the functions of most lncRNAs remain elusive due to the large number of annotated sequences and secondary structures. LncRNAs are defined as a type of RNA transcript greater than 200 nt in length that has no apparent coding potential. Dysregulation of lncRNAs is often associated with pathological processes, including cancer, leukemia, Alzheimer’s disease and diabetes [23, 24]. Recently, it has also been reported that lncRNAs are involved in regulating endothelial cell function [25–28]. In addition, lncRNA myocardial-infarction-associated transcript (MIAT) and lncRNA nuclear paraspeckle assembly transcript 1 (NEAT1) could improve endothelial barrier function [29, 30]. In this study, we confirmed that multiple dysregulated lncRNAs were found during the process of endothelial barrier dysfunction upon exposure to PM2.5. Our results further confirmed the regulatory role of lncRNAs on endothelial barrier function.

Studies have demonstrated that lncRNAs play important regulatory roles in the mammalian system by regulating gene expression through various mechanisms, including chromatin modification, posttranscriptional regulation of gene expression (such as splicing, mRNA translation and mRNA degradation) and acting as a ceRNA sponge of miRNAs [29–34]. Reports have indicated that miR-21 can be regulated by lncRNAs in various pathological processes [35–38]. It has been shown that GAS5 and miR-21 are likely in the same AGO2 complex, and GAS5 could act as a ceRNA to sponge miR-21 in breast cancer cells [35]. In the course of our research, reports demonstrated that lncRNA CASC2 sponges miR-21 in oral squamous cell carcinoma and in the modulation of sensitizes cervical cancer to cisplatin, and lncRNA FTX can also negatively control miR-21-5p during status epilepticus [36–38]. Our previous report demonstrated that miR-21-5p transcription is regulated by STAT3 and is involved in PM2.5-induced vascular barrier dysfunction [6]. According to the reports indicated above, we further identified potential lncRNAs that could regulate the expression of miR-21-5p during endothelial cell permeability increase upon exposure to PM2.5. The lncRNA HCG18 has been reported to regulate gene expression by acting as a ceRNA that sponges miR-34c-5p in intervertebral disc degeneration or miR-146a-5p in bladder cancer cells [11, 17]. In this study, we confirmed that HCG18 is the most downregulated lncRNA that could bind to miR-21-5p in PM2.5-treated HUVECs. In addition, HCG18 could act as a ceRNA to negatively regulate the expression of miR-21-5p. Combined with previous reports, our results further demonstrated that a single miRNA could be regulated by multiple lncRNAs.

Sex-determining region Y-box 7 (SOX7) is a member of the SOXF subfamily, which belongs to the high mobility group (HMG) superfamily of transcription factors [39]. It has been reported that SOX7 can be expressed in vascular endothelial cells and plays an important role in angiogenesis and vascular development [19, 40–43]. However, whether SOX7 participates in maintaining vascular endothelial barrier function is still unclear. SOX7 can bind to the promoter region of VE-cadherin and transcriptionally regulate VE-cadherin expression in the hemogenic endothelium during hematopoietic development [19]. In addition, our previous report indicated that exposure to PM2.5 in HUVECs can down-regulate the expression of VE-cadherin and further increase endothelial permeability [6]. These studies suggest that SOX7 might be involved in endothelial barrier function through transcriptional regulation of VE-cadherin expression. In this study, we confirmed the hypothesis that inhibiting SOX7 can decrease the expression of VE-cadherin and further increase endothelial cell permeability upon exposure to PM2.5 in HUVECs. Although SOX7 acts as a transcription factor to regulate gene expression, the regulatory mechanism of PM2.5-induced down-regulation of SOX7 in HUVECs is still unknown. It has been reported that SOX7 can be regulated by miRNAs in hepatocellular carcinoma and glioblastoma [44, 45]. Our work demonstrated that miR-21-5p can directly bind to the 3’UTR of SOX7 and post-transcriptionally regulate SOX7 expression in HUVECs. While our research was conducted, it was reported that miR-21-5p regulates SOX7 expression in a rat temporal lobe epilepsy model [36]. Therefore, we further confirmed that SOX7 is a target of miR-21-5p and that the miR-21-5p/SOX7 axis plays an important role in maintaining endothelial barrier function.

In summary, this study demonstrates that down-regulation of lncRNA HCG18 in HUVECs is involved in PM2.5-induced vascular barrier dysfunction. These findings further confirm the molecular mechanism by which miR-21-5p inhibits SOX7 and suppresses VE-cadherin expression, which was not clearly understood in our preliminary report.

## MATERIALS AND METHODS

### Regents and antibodies

Rabbit anti-SOX7 (BA3297-2, diluted at 1:1000 for WB (western blotting assay)) was obtained from Boster (Wuhan, China), rabbit anti-VE-cadherin (#2500, diluted at 1:1000 for WB and 1:200 for IF (immunofluorescence staining) and rabbit anti-GAPDH (#5174S, diluted at 1: 1000 for WB) were obtained from Cell Signaling Technology, Inc. (Danvers, MA, USA). MiR-21-5p mimics (miR10000076), miR-21-5p inhibitor (miR20000076) were purchased from RiboBio Co., Ltd., China.

miR-21-5p mimics are chemically synthesized miRNA which is easy to control transient transfection into the cell, while miR-21-5p inhibitor is single-stranded synthetic inhibitor having complementary sequences to target miRNA.

PM2.5 were sampled from Beijing and analyzed as our previous research [7].

### Cell lines

Human umbilical vein endothelial cells (HUVECs) were isolated from human umbilical veins provided by Affiliated Hospital of Guangzhou Medical University, according to previous report [46]. The isolated cells were maintained in endothelial cell growth medium EBM-2 (CC-3162, Lonza). 293T cells were kindly provided by the Stem Cell Bank, Chinese Academy of Sciences, and cultured in Dulbecco's Modified Eagle Medium (C11995500BT, GIBCO) with 10% FBS (Gibco), penicillin (100 U/mL) and streptomycin (100 mg/mL). All the cells were incubated in 5% CO_2_ incubator at 37°C.

### Establishment of cell model

HUVECs were treated with PM2.5 working solution (80μg/mL) for 24 hours, which is diluted from a stock solution (10mg/ml) with EBM-2 medium.

### Microarray analysis

Total RNAs were extracted from PM2.5-treated or PM2.5-untreated HUVECs (n=3). The differential expression of lncRNAs was detected using the RiboArray® human lncRNA microarray according to RiboBio lncRNA Hybridization protocol.

### Real-time quantitative PCR (qRT-PCR)

Total RNAs were extracted from HUVECs using NucleoZol (MACHEREY-NAGEL, Germany) and reverse transcribed using PrimeScript^TM^ RT Reagent Kit (RR014A, TaKaRa, Japan) and the real-time PCR analysis was carried out using SYBR® Premix ExTaq™ II (TliRNaseH Plus) PCR Kit (RR820A, TaKaRa, Japan) on the 7500 Real-Time PCR System (ABI). The PCR primers were designed and synthesized by RiboBio Co., Ltd., China. The results were analyzed using the 2^–ΔΔCT^ method. The expression of U6 was selected as a reference.

### RNA immunoprecipitation (RIP)

RIP assay was performed using Magna RIP^TM^RNA-Binding Protein Immunoprecipitation Kit (Millipore, MA, USA). HUVECs were lysed in the lysis buffer and incubated with RIP buffer containing magnetic beads that coated with anti-human argonaute 2 (AGO2) antibodies (Millipore, USA) or IgG (Millipore, USA) at 4°C overnight. RNAs were purified from RNA-protein complexes bounded to the beads. Then, the expression of HCG18 and miR-21 was detected by qRT-PCR.

### Plasmid construction and cell transfection

(1) For expression of HCG18 or SOX7, the full-length HCG18 sequence and full-length coding sequence (CDS) of SOX7 were amplified from human cDNA extracted from HUVECs and cloned into pcDNA3.1(+) vector (Invitrogen, Carlsbad, CA).

(2) The binding sites of HCG18 and miR-21-5p were predicted by Star base v2.0, RNA22 and miRDB. Then, a 500 bp wild-type (WT) DNA sequence containing the putative binding sites and its mutant binding sites were amplified and cloned into psiCHECK2 vector (Promega, Madison, WI) separately. The binding sites of miR-21-5p on SOX7 ’UTR was predicted by TargetScan7.2. A 100-nt wild-type (WT) DNA sequence containing the putative binding sites and its mutant binding sites (MUT) for miR-21-5p in the SOX7 3’UTR was amplified and cloned into psiCHECK-2 vector separately.

(3) For promoter analysis, a 400-bp genomic fragment (-552~+63) containing the predicted wild-type (WT) SOX7 binding sites and its mutant binding sites (MUT) in VE-cadherin promoter were amplified and cloned into pGL3-Basic vector (Promega, Madison, WI). All primers are listed in Table 1.

**Table 1:** Primers used in polymerase chain reaction (PCR) assay.

**Gene**		**Primer sequence (5’→3’)**
lncHCG18 full length on Plasmid	Forward	GAATTCTTAGAGGATCCTGTTT
	Reverse	TCTAGATTTTTAGAAAGATGAT
SOX7 CDS full length on Plasmid	Forward	CTGCTGGGAGCCTACCCTTGG
	Reverse	CAGCCAGGACGGAGATGAG
lncHCG18-244	Forward	GAATTCTTAGAGGATCCTGTTT
	Reverse	TCTAGATTTTTAGAAAGATGAT
SOX7 3’UTR	Forward	GTTGATGATATGATTGACTGATGC
	Reverse	CTGTTCCAAAGTATGAGTTGTTCT
SOX7 3’UTR mut	Forward	CATAAAACAGTATCACAGACATGCCGTGGAACTC
	Reverse	TTCAAAAAAACGAAACAGTTCCACGGCATG
VE-Cadherin promoter	Forward	AGGAGGGTTAAATGTGATGCTG
	Reverse	GGGATGTTTCTGTTCCGTTG
VE-Cadherin promoter mut	Forward	CAAAGGCGACCCAACAGGAA
	Reverse	CCTGTTGGGTCGCCTTTGT

### Luciferase activity assay

HCG18 3’UTR reporter vectors and miR-21-5p mimics, SOX7 3’UTR reporter vectors and miR-21-5p mimic, SOX7 expression vector and VE-cadherin promoter reporter vectors were cotranfected into 293T cells separately. Then, luciferase activity was measured at 48 h post transfection using the Dual-Luciferase Reporter Assay System (E2920, Promega, USA). Renilla luciferase activity was normalized to firefly luciferase expression.

### Western blotting assay

Total proteins were extracted from HUVECs using RIPA lysis buffer (WB-0072, Dingguo) with PMSF (WB-0072, Dingguo). Protein concentrations were determined by Mithras LB940 (BERTHOLD, Germany). Total proteins were separated using 10% (w/v) Sodium dodecylsulphate-polyacrylamide gel electrophoresis (SDS-PAGE), and then transferred onto a polyvinylidene fluoride (PVDF) membrane (Millipore, MA, USA). The membranes were blocked using 5% (w/v) non-fat powder milk, and incubated with relative primary antibody and horseradish peroxidase (HRP)-conjugated secondary antibodies. The protein bands were further visualized using the Image Quant LAS 4000 system (GE Healthcare, Waukesha, WI, USA) and quantified using Quantity-One protein analysis software (Bio-Rad Laboratories, Hercules, CA, USA). GAPDH was used as a reference.

### *In vitro* FITC-dextran transwell assay

HUVECs were seeded on the top chamber of transwell inserts. FITC-dextran was added to the top chamber and samples were removed from the bottom chamber after 5 h. Then, the fluorescence intensity was analyzed with a fluorometer at an excitation of 490 nm and an emission of 530 nm.

### Immunofluorescence assay (IF)

HUVECs was on the coverslips and transfected with the expression plasmids of HCG18 or SOX7 or miR-21-5p mimics, and then treated with PM2.5. Cells were fixed in 4% polyoxymethylene and permeated in 1% Triton X-100. Then, cells were blocked using 1% BSA and incubated with rabbit anti-VE-cadherin antibody and Alexa Fluor 488 donkey anti-rabbit IgG (H+L) (Life Technology, USA). Following it, cells were stained with DAPI and photographed using a Laser scanning confocal microscope (Axio-Imager_LSM-800, ZEISS).

### Database or software

RNA22 (version 2.0, http://cm.jefferson.edu/rna22/Interactive/) was used to predict binding sites between lncRNA and miRNA.

TargetScan (ver 7.2, http://www.targetscan.org/vert_72/) was used to predict binding sites between miR-21 and SOX7 3’UTR region.

JASPAR (http://jaspar.genereg.net/) was used to predict binding sites between SOX7 and VE-Cadherin promoter region.

### Statistical analysis

All data are presented as the mean ± standard deviation (S.D.) from 3 independent experiments. Statistical analysis was using SPSS 16.0 software (SPSS, Chicago, IL, USA) and GraphPad Prism 5 (GraphPad Software, Inc., San Diego, CA, USA). Differences between groups were analyzed using a two-sided Student’s *t*-test. *P*<0.05 were considered statistically significant.
